# A Queer Matrimonial Mart

**Published:** 1892-03

**Authors:** 


### A QUEER MATRIMONIAL MART.

In alluding to the social evil which has cast its baneful shadows over every land from the earliest ages, Dr. Coltman, author of recent work on the Chinese of North China, has given us an account of a highly original mode of suppressing it, adopted by a magistrate of Chinanfu, which we cannot do better than to give in the author's own words. It is as follows, and if it may not furnish a precedent for our local magistracy, we can do no less that commend the motive and originality of the scheme.

In the early seventies a Judge Yuan occupied the magistracy of Chinanfu, who will be long remembered by the people of that district. This judge, although a heathen, or, more correctly speaking, a Confusianist, was a good and just man. As soon as appointed judge, he ordered all gambling houses and places of prostitution to close their doors, and gave a few days only to the constables to carry the order into effect. When the time had expired, he sent out his police and had all the prostitutes in the city arrested and brought into his yamen-yard. An eye witness of the scene assured me that the yard, which is by no means a small one, was full, girls of twelve, up to women of forty and over, some gaily painted and well dressed, some in rags, many with eyes heavy from large doses of opium—for nearly all the lost women take to the drug as a dissipation and a means to relieve pain.

The judge then issued a proclamation declaring the character of the women and that he hoped to make honest wives of them by selling them off at auction the following day. Any farmer or business man who was unmarried, would be allowed to purchase one. No one was allowed to buy more than one. A great crowd attended the sale, some to buy a wife, but most, of course, to witness the proceedings. At the hour named, the judge and his chief of police appeared, and after repeating a declaration of the character of the women, said they would not sell to the highest bidder, as at first proposed, but that each man in the audience desiring to purchase a wife, could select his bargain from the many in the yard, and she would be sold by weight. For some time no one would pick out one ; but at last an old farmer, who felt time to be precious, walked up and picked out a woman some forty years of age, who was stout and hearty. "Weigh her," said the judge ; and immediately screaming and kicking, she was hoisted in the balance. "Ninety cutties," said the weigher. "But how much a cutty ?" said the old farmer, rather scared lest his money was insufficient. Curiously enough, nothing had been said about the price per cutty. The old judge was a shrewd man, and fond of a joke, withal ; so he immediately asked, "What is the price of pork to day ?" "Ninety cash a cutty," replied the auctioneer. "Then sell them at ninety cash," ordered the judge. After this the sale waxed fast and furious, and by night the last one was off. One young farmer, having bought one of the younger women, seated her on his wheelbarrow and wheeled toward home for a distance of three miles, when his purchase complained of feeling sick, and, on his asking what was the trouble, she told him she was accustomed to using three drahms of opium daily, and that she would die if she did not obtain some speedily. The poor farmer was sadly perplexed, as well as frightened, but immediately wheeled his purchase back to the sale and requested to see the auctioneer.

At first no one would listen to him ; but the judge, who personally superintended the entire day, observing some trouble on the border of the crowd, soon had the man brought before him. "What is the matter, my man ?" he kindly inquired. The poor fellow fell on his knees, and, striking his head in the dust again and again, said : "Your honor, the woman I bought will ruin me ; so I have brought her back." "How ruin you ?" inquired the judge. "Why she takes three drahms of opium daily, your honor, and my earnings altogether are not enough to buy her opium," replied the trembling countryman. "Then she won't do for you," said the judge : "but see if you cannot find one who does not use the drug, and if you do succeed, have her weighed. If she is heavier, pay the difference in the weight, and lighter, you will be refunded ; how will that do ?" And the judge smiled to see the expression of intense relief visible on the young man's countenance. The delighted farmer hastened to do as told, and secured a woman who professed to be free of the habit, and who was only too glad to leave her former condition to become the wife of an honest farmer.

